# The Mediterranean Mussel *Mytilus galloprovincialis* (Mollusca: Bivalvia) in Chile: Distribution and Genetic Structure of a Recently Introduced Invasive Marine Species

**DOI:** 10.3390/ani14060823

**Published:** 2024-03-07

**Authors:** Pablo A. Oyarzún, Jorge E. Toro, José J. Nuñez, Gonzalo Ruiz-Tagle, Jonathan P. A. Gardner

**Affiliations:** 1 Centro de Investigación Marina Quintay (CIMARQ), Universidad Andrés Bello, Quintay 2340000, Chile; gjruiztagle@uc.cl; 2 Instituto de Ciencias Marinas y Limnológicas (ICML), Facultad de Ciencias, Universidad Austral de Chile, Independencia 631, Valdivia 5090000, Chile; jtoro@uach.cl (J.E.T.); jjnunezn@gmail.com (J.J.N.); 3 School of Biological Sciences, Victoria University of Wellington, P.O. Box 600, Wellington 6140, New Zealand; jonathan.gardner@vuw.ac.nz

**Keywords:** biodiversity, bioinvasion, blue mussels, genetic diversity, mitochondrial DNA, *Mytilus edulis* species complex, non-native species, south-eastern Pacific Ocean

## Abstract

**Simple Summary:**

The genetic pool of invasive species greatly influences their ability to establish and spread. The blue mussels *Mytilus galloprovincialis*, originating from the Mediterranean, are major invaders worldwide. Our study examined their genetic diversity and structure in Chile, comparing them with native populations. We used mitochondrial DNA to analyze the genetic traits and origins of invasive populations. Two lineages of *M. galloprovincialis* were identified, i.e., NW Atlantic and Mediterranean. Although recently introduced, no genetic structure was found in Chilean populations. However, a mixture of lineages increased the genetic diversity along the coast. The invasion coastal extent remains small (~100 km), suggesting their recent introduction. The expansion of these populations may encounter natural barriers; nevertheless, it is advisable to monitor these species’ distribution.

**Abstract:**

The genetic characteristics of invasive species have a significant impact on their ability to establish and spread. The blue mussel (*Mytilus galloprovincialis*), native to the Mediterranean Sea, is a leading invasive species of intertidal coasts throughout much of the world. Here, we used mitochondrial DNA sequence data to investigate the genetic diversity and phylogeographic structure of invasive (*M. galloprovincialis*) versus native (*Mytilus chilensis*) populations of blue mussels in Chile. We evaluated whether genetic diversity in invasive populations could be explained by the genetic characteristics of the native sources from which they might be derived. A phylogenetic analysis confirmed two lineages of the invasive *M. galloprovincialis*, i.e., the NW Atlantic and the Mediterranean lineages. We found no evidence of genetic structure in the invasive range of *M. galloprovincialis* in Chile, most probably because of its recent arrival. We did, however, detect a spatial mixture of both *M. galloprovincialis* lineages at sampling locations along the Chilean coast, giving rise to higher levels of genetic diversity in some areas compared to the population of native *M. chilensis*. The coastal area of the invasion is still small in extent (~100 km on either side of two large ports), which supports the hypothesis of a recent introduction. Further expansion of the distribution range of *M. galloprovincialis* may be limited to the north by increasing water temperatures and to the south by a natural biogeographic break that may slow or perhaps stop its spread. The use of internal borders as a tool to minimise or prevent *M. galloprovincialis* spread is therefore a genuine management option in Chile but needs to be implemented rapidly.

## 1. Introduction

Biological invasions are now very frequent [1,2], largely because of globalisation that has increased ship transport and associated accidental transfers of species and also because of the deliberate introduction via human-mediated activities such as agriculture and aquaculture [3,4,5]. Biological invasion now represents one of the most important environmental and conservation challenges and needs to be addressed urgently to help us understand the complex events underlying it in a changing world [6]. An introduced species with biologically competitive capabilities and that lacks natural enemies may become established and cause significant detrimental effects to native species [7,8,9] and/or their habitat [10,11,12]. However, even though there have been several successful efforts to trace and understand the mechanisms of invasion (e.g., [5,13,14,15]), the biological components of invasions and how they interact to contribute to invasion success or failure in many systems are still not well understood [16,17,18].

In the understanding of how and why some invasions succeed and others fail, in terms of either initial establishment or multi-generational longevity, the role of genetic diversity has been a focus of attention. Typically, the genetic diversity of the founder (invasive) population is thought to be positively related to its probability of invasion success [19,20]. However, whilst genetic diversity of the invading species is known to be important in certain cases, the role of genetic diversity as a contributor to a successful invasion is far from clear for many or all species [12,18,21,22,23]. When the introduction of a non-native species occurs, a genetic bottleneck may be generated because the small number of individuals of the founder population are likely to have reduced genetic diversity compared to its (very large) native source population [24,25]. This population genetic bottleneck (founder effect) may reduce the adaptation potential of the introduced individuals in new habitats because the invaders have only a subset of the source individuals’ genetic diversity [19,20,26]. For the same reason, introductions may be subject to a high risk of inbreeding, further reducing the adaptive potential, limiting the reproductive success (via inbreeding depression) and increasing the probability of extinction of the invaders [27,28]. Nonetheless, many introduced species establish and thrive despite the small pool of founder individuals and the likelihood that they are characterised by reduced genetic variation compared to source populations (e.g., [18,19,22,23]), whilst others show a limited establishment presence or a reduced invasion success (e.g., [29,30]). In the face of uncertainty about just how important genetic diversity amongst invaders is for promoting invasion success, it is important to study the genetic patterns of non-native species that may exhibit different invasive scenarios.

*Mytilus galloprovincialis* Lamarck, 1819, is a smooth-shelled blue marine mussel (Family Mytilidae) that inhabits the rocky intertidal zone and, in some areas, the shallow subtidal zone. It is ecologically important in terms of both biomass and areal coverage and plays a key role in the energy transfer of primary production from coastal waters to the shore (e.g., Gardner et al., 2016 [7]; 2021 [31] and references therein). *M. galloprovincialis* belongs to the *Mytilus edulis* species complex (*M. galloprovincialis*, *M. edulis* Linné, 1758, *M. trossulus* Gould, 1850, *M. planulatus* Lamarck, 1819, *M. chilensis* Hupé, 1854, and *M. platensis* d’Orbigny, 1842; [9,31,32,33,34,35,36,37,38]), which is composed of morphologically cryptic but genetically distinct species [31,39]. These species inhabit all oceans, whilst also exhibiting an antitropical distribution [40]. *M. galloprovincialis* is the most widely distributed species and is now found in all continents. However, it is considered to be endemic to the Mediterranean Sea and parts of the coastline of the NW Atlantic Ocean (two distinct lineages have long been known, but both are recognised as being *M. galloprovincialis*; Popovic et al., 2020 [41]; Gardner et al., 2021 [31] and references therein) and, for that reason, is known as the Mediterranean mussel [32,42].

The Mediterranean mussel has become successfully established in many countries and is considered one of the 100 most invasive species in the world [43,44]. During the last century, this bivalve mollusc successfully invaded the marine coastal habitats of both hemispheres, including locations in Hong Kong, Japan, the west coast of North America and, in the Southern hemisphere, South Africa, parts of Australia, New Zealand and locations in South America [7,8,9,31,33,35,41,45,46,47,48,49,50,51]. The sites at which this mussel has been reported are often associated with ports and routes of transoceanic navigation; so, it is assumed that ballast water and/or hull fouling are likely to be the main vectors for the translocation of this mussel [7,14,52,53,54,55].

In locations where the invasive *M. galloprovincialis* establishes, it often covers and takes over the available substratum and rapidly displaces (out-compete) native species [8,56,57], causing economic and ecological damage [7,31,58]. However, whilst the invasion process of *M. galloprovincialis* has been successful in most cases (that we know of), it has been unsuccessful in a limited number of others. For example, the most dramatic case was reported from South Africa, where *M. galloprovincialis* was first detected in Saldanha Bay at the beginning of the 1970s (Grant and Cherry 1985 [49]; reviewed by Ma et al., 2021 [59]). The identification of the invader was easy, given that South Africa has no native members of the genus *Mytilus* [49]. A few years later, *M. galloprovincialis* had extended its distribution very rapidly towards the north at an average speed of 115 km y^−1^ and to the south at about 25 km y^−1^ [10]. The invasion now spans ~2800 km of coastal area, having resulted in the partial replacement of endemic mussels (i.e., *Perna perna* (Linnaeus, 1758), *Aulacomya atra* (Molina, 1782) and *Choromytilus meridionalis* (Krauss, 1848)), making *M. galloprovincialis* the main intertidal organism in several rocky coastal areas to the west of South Africa and in Namibia [58,59,60]. *M. galloprovincialis* is now considered the most successful invasive marine species in the south of Africa [61]. Nonetheless, Robinson et al. [58] reported the complete invasion collapse of *M. galloprovincialis* at one highly sedimented location in South Africa and the subsequent (short-term) recovery of the native biota. This example of invasion collapse is important for *M. galloprovincialis* because there are very few such documented cases (Gardner et al., 2021 [31] and references therein). The cause of the collapse remains unknown but is most probably linked to the highly sedimented nature of this particular site [58].

A very different scenario from that described for South Africa has been observed in South America, where native blue mussels are found extensively on both the Atlantic (*M. platensis*) and the Pacific (*M. chilensis*) coasts [9,33,35,36,37,62,63]. The presence of the invasive Mediterranean mussel was first reported from southern central Chile (Dichato) by Daguin and Borsa (2000) [47] in a sample collected in 1998 and then more widely from the Región del Bio Bio, Chile (~36° S) [64]. The uncertainty of the invasion history in Chile (and elsewhere) stems in large part from the lack of morphologically distinct features between *M. galloprovincialis* and the native *M. chilensis*, which resulted initially in the erroneous identification of the invasive species as its native counterpart [65]. With the use of molecular markers, it has been possible to understand, at least in part, the biogeography of these mussels on the Chilean coast [9,33,35,50,66,67]. However, there is surprisingly little knowledge about the spread of *M. galloprovincialis* in Chile or the genetic diversity of this non-native species, despite its reputation as a highly successful invader and the fact that it can interbreed with native *M. chilensis* (reviewed by Gardner et al., 2021 [31]). There has been only limited study of the possible range expansion of this non-native species [68] and no study of the effects of its ecological interaction with the endemic mussel *Mytilus chilensis* along the Chilean coast. It is, however, known that these two species are able to hybridise and produce viable larvae, juveniles and adults [63,69] and that mixed-ancestry individuals are often reported as occurring in the wild (e.g., [9,33,51]). Interestingly, it was observed that *M. galloprovincialis* has a very limited distribution along the Chilean coast [67,68], despite its first record in 1998 [47] and its well-documented history of establishment success elsewhere, followed by rapid range expansion [70]. This situation is somewhat surprising because *M. galloprovincialis* has been interacting with native Chilean mussels (i.e., *Choromytilus chorus* (Molina, 1782) and *Aulacomya atra*) that have similar characteristics to those of mussels found in South Africa (i.e., *Perna perna*, *Aulacomya atra* and *Choromytilus meridionalis*) where *M. galloprovincialis* has had a major ecological impact (Zardi et al., 2018 [61] and references therein). The question, therefore, from the Chilean example is why is the spread of *M. galloprovincialis* so limited compared to other regions and what explains it?

An understanding of the ecological and evolutionary processes that promote invasion success (i.e., establishment and subsequent spread) is of fundamental importance to develop approaches in the long term to prevent future invasions and to manage the existing status [7,9,15,41,71]. However, most studies have focussed on successful invaders and not on species that failed to invade or that became established but whose range of distribution has not expanded. The unsuccessful invader examples may be the key to understand the importance of genetic diversity and therefore to predict the success of an invasion [29,30]. There is therefore a need for studies that consider cases of successful, limited, and unsuccessful invasions to determine the genetic mechanisms that underlie the success or failure of invasive species. In the present study, we used phylogenetic approaches and population genetics analyses based on sequences of mitochondrial DNA (mtDNA) to characterise the genetic patterns of *M. galloprovincialis* and *M. chilensis* populations sampled from Chile, Spain and South Africa. The objective was to clarify the genetic structure of the endemic and exotic species on the Chilean coastline to better understand the potential factors that drive their patterns of spatially explicit genetic variation and how these factors may contribute to their invasion success and further spread beyond the initial point of establishment.

## 2. Materials and Methods

### 2.1. Sampling, DNA Extraction, Amplification and Sequencing

**Figure 1:**
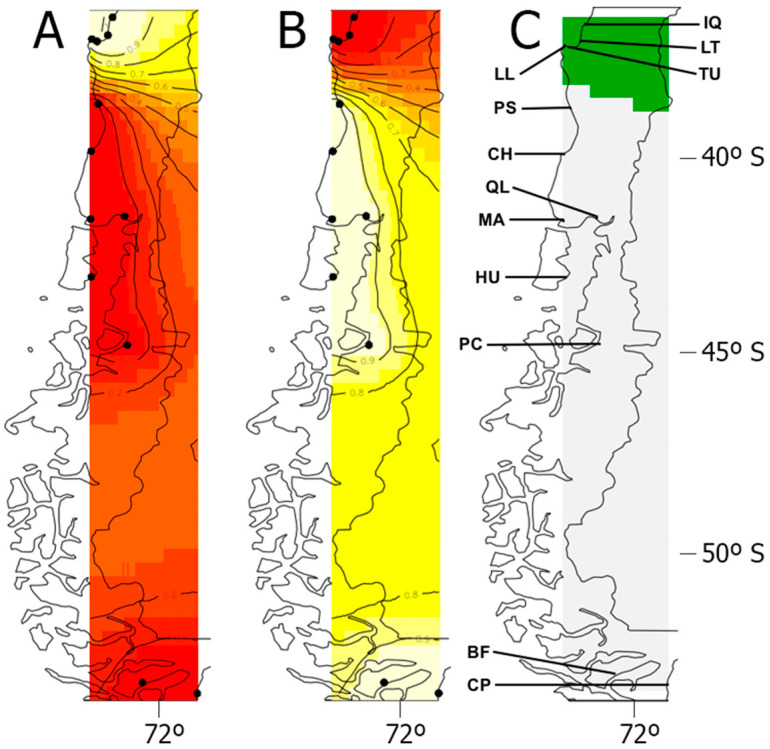
Geneland result for *K* = 2 groups using the spatial model with uncorrelated allele frequencies (data COI). Plots representing the assignment of pixels to the northern (**A**) and southern (**B**) groups in Chile; (**C**) map of estimated posterior probability of population membership (by posterior mode), that is, the two groups shown in green and grey. For plots A and B, the highest membership values are in light yellow, and the contour lines indicate the spatial position of genetic discontinuities between populations. Site information is reported in Table 1 (*M. galloprovincialis*: IQ, LT, LL, TU; *M. chilensis*: PS, CH, MA, QL, HU, PC, BF, CP).

To better understand the distribution of *Mytilus* spp. in Chile, 26 sites with rocky substrates were surveyed. Blue mussels were found at only 12 sites (Table 1 and Table S1). Mussels, both invasive *Mytilus galloprovincialis* and native *M. chilensis*, were sampled at 4 and 8 sites, respectively, along the coast of Chile (Figure 1; Table 1). We did not sample sites with mixed species. In addition, locations were sampled in Spain (native *Mytilus galloprovincialis*) and South Africa (introduced *Mytilus galloprovincialis*) (Table 1). Because of doubly uniparental inheritance, only female individuals were used in this study. All samples were collected alive from intertidal and subtidal beds. Immediately after collection, a 1 cm^2^ piece of tissue was excised from the mantle tissue of each individual, fixed in 95% ethanol and stored at 4 °C before DNA extraction. Total genomic DNA was extracted using the genomic E.Z.N.A.^®^ Tissue DNA kit (Omega Bio-Tek, Norcross, GA, USA).

Our first step was to make a taxonomic determination of the species using the RFLP assay described by Santaclara et al. (2006) [72], which is based on the partial digestion of the glu gene with the restriction enzyme *Aci I*. This allows the identification of *M. chilensis*, *M. edulis*, *M. galloprovincialis* and *M. trossulus*.

Our second step was to amplify fragments of the COI and 16S genes using the universal primers LCO1490/HCO2198 [73] and 16SAR/BR [74], respectively. Amplifications were performed in a 25 µL reaction volume consisting of 2.5 µL of 10× buffer (50 mM KCl, 10 mM Tris HCl, pH 8.0), 1.0 µL of 50 mM MgCl_2_, 200 mM dNTPs, 0.5 µL of each primer (10 pg/µL), 1 U of Taq (Invitrogen^TM^, Carlsbad, CA, USA), 17.5 µL of double-distilled water and 20 ng of DNA. The thermocycling parameters included an initial denaturation step at 94 °C for 3 min, followed by 35 cycles at 94 °C for 1 min, 48 °C (COI) and 51.5 °C (16S) for 45 s, and 72 °C for 1 min and ended with a final 6 min extension at 72 °C. All PCR and restriction products (see below) were examined in 2% agarose gels stained with SYBR^®^ Safe DNA and photographed under a blue light transilluminator (Invitrogen^TM,^ Carlsbad, CA, USA). For every gel, the size of the amplified fragments was estimated using a 100 bp DNA ladder (Invitrogen^TM^, Carlsbad, CA, USA).

The DNA products were purified and sequenced in both directions. The sequences were edited using Geneious^®^ v.11.0.4 (https://www.geneious.com/). All alignments were performed in MAFFT v.7 [75] under the iterative method of global pairwise alignment (G-INS-i) [76], and default settings were chosen for all parameters.

### 2.2. Data Analyses

#### 2.2.1. Population Genetic Structure

The spatially explicit Bayesian clustering program Geneland v.3.2.4 [77] (an extension of program R 3.1.2. [78]) was used to investigate the geographic genetic structure along the coast of Chile, using the COI data. We converted variable base sites into bi-allelic (allele-like) data, so that the COI input file was a binary file, using PGDSpider v.2.1.1.1 [79]. We ran ten independent runs, where the parameters for possible populations were *K* = 1–12, and the number of MCMC iterations was 1,000,000, saving every 100 steps (Figure S1). A “burn-in” of 2000 generations was trimmed. A contour map of the posterior mode of population membership was drawn to visualise the genetic substructure within the study site.

#### 2.2.2. Gene Lineages, Genetic Distance and Demographic Analyses

The genealogical relationships of *M. galloprovincialis* in Chile were assessed by estimating the frequency of haplotypes at each site using DnaSP v.5.1 [80].

The evolutionary divergence amongst the groups (*Mytilus galloprovincialis* from Chile, Europe and South Africa) was estimated in MEGA v.7 [81]. Standard error estimates were obtained by a bootstrap procedure (1000 replicates). The analyses were conducted using the Tamura–Nei model [82], which was estimated in PartitionFinder v.2.1.1 [83] for the sequence data. The rate variation among the sites was modelled with a gamma distribution (shape parameter = 1).

#### 2.2.3. Population Genetic Diversity

From the COI and 16S sequences, standard diversity indices such as the number of mitochondrial haplotypes (K), haplotypic diversity (H), the number of polymorphic sites (S), the mean number of pairwise differences (Π), as well as nucleotide diversity (π), were estimated for each population using DnaSP v.5.1 [80].

#### 2.2.4. Demographic Analyses—Chile

For mussels from Chile only (*M. chilensis* and *M. galloprovincialis*), to measure the deviation from the null hypothesis of a constant population size and random mating, neutrality testing of COI and 16S sequences was conducted in DnaSP v.5.1 and Arlequin v.3.5 software. First, the Fu’s *F*_s_ [84] and Tajima’s *D* [85] values were estimated by comparing the differences between the number of segregating sites and the average number of nucleotide differences. Positive values indicated a lack of significant recent mutations that might have resulted from balancing selection, population structure or a decline in population size. Negative values reflected excesses of recent mutations that might indicate population expansion or selective sweeps. Second, mismatch distribution analysis was carried out in Arlequin v.3.5 [86], comparing the frequency distribution of pairwise differences among haplotypes against a model of rapid expansion [87]. The expected distributions were generated by bootstrap resampling (10,000 replicates) using a model of sudden demographic expansion. The sum of square deviations and the raggedness index between the observed and the expected mismatch were used as test statistics; *p*-values were calculated as the probability of simulations producing a greater value than the observed value.

An independent measure of demographic history was estimated using a Bayesian Markov chain Monte Carlo (MCMC) coalescent approach implemented in BEAST v.1.8.1 [88]. The Bayesian skyline plot (BSP) uses MCMC sampling procedures to estimate a posterior distribution of effective population size through time from a sample of gene sequences, given a previously specified nucleotide substitution model [89,90]. The time dimension of the analyses was calibrated by fixing an evolutionary rate of 9.51 × 10^−8^ calculated for the COI gene in the genus *Mytilus* [91]. The prior on this rate was set to follow a normal distribution (lognormal relaxed clock) allowing for uncertainty around the estimate. The analyses were run using a best-fit model of evolution identified from the BIC value [92] using PartitionFinder v.2.1.1 [83]. Subsequently, 100 million MCMC generations were sampled every 1000 generations and launched from a random starting tree. The analysis was repeated in triplicate, and log files were examined using Tracer v1.5 [93] to determine the appropriate burn-in (25% of the chain length) and ensure that the runs were returning samples from the same distribution and that the effective sample sizes for all demographic statistics were >200. The post-burn-in log and tree files from each independent run were combined using LogCombiner v.1.8.1, and the trees in the posterior sample were summarised by Bayesian skyline reconstruction using a stepwise skyline variant.

#### 2.2.5. Phylogenetic and Phylogeographic Analyses

To elucidate the intraspecific phylogeny of *M. galloprovincialis*, phylogenetic trees were constructed using maximum likelihood (ML) and Bayesian inference (BI) approaches. Evolutionary models and partitioning strategies were evaluated with PartitionFinder v.2.1.1 [83], which chose the best partition from the BIC values [92]. An ML tree was inferred using GARLI v.2.0 [94], with branch support being estimated by nonparametric bootstrapping (200 replicates; above this value, the output results did not fluctuate). Bayesian analyses were performed using MrBayes v.3.2 [95]. Each Markov chain was started from a random tree and run for 15 × 10^6^ generations with every 1000th generation sampled from the chain. Stationarity was checked as suggested in Nylander et al. (2004) [96]. All sample points prior to reaching the plateau phase were discarded as “burn-in”, and the remaining trees were combined to find the a posteriori probability of phylogeny. The analyses were repeated three times to confirm that they all converged on the same results. Posterior probability values > 0.90 were taken as statistical support for a clade being present in the true tree [97]. For this analysis, the sequence data of *M. galloprovincialis* from Chile (Table 1) and sequence data from GenBank (Table S2) were used.

A discrete phylogeographic analysis was performed to reconstruct the ancestral population of *M. galloprovincialis*. For this, a Bayesian analysis implemented in BEAST v.1.8 was used [93]. The analysis included all available COI gene sequences (from Spain, South Africa, Italy, Greece and Chile; see Table 1 and Table S2). We modelled each locality as a discrete trait using a symmetric substitution model inferring the social network with a Bayesian stochastic search variable selection procedure (BSSVS). The ancestral state of all the ancestors was reconstructed. We used an uncorrelated relaxed clock model with lognormal distribution and a clock mean rate of 9.51 × 10^−8^ estimated for the COI gene in *Mytilus* Linnaeus, 1758 [91]. A coalescent tree prior with constant population size was used [98]. The analysis was run for 7 × 10^7^ iterations, sampling every 5000 generations. The stationarity (effective sample size values > 200) of the run was verified with Tracer v1.5 [93]. The first 25% of the samples were discarded (burn-in). Then, a consensus was built with TreeAnnotator v.1.8.1 using the maximum clade credibility method.

## 3. Results

No blue mussels were found at latitudes lower than 36° S on the Chilean coast. In addition, *Mytilus* spp. did not show a continuous distribution across the sampled sites (Table 1 and Table S1).

### 3.1. Sequence Characteristics

The sequence data (COI = 676 bp; 16S = 470 bp) from 120 mussels of the species *Mytilus galloprovincialis* from Spain (native), South Africa and Chile (for both, introduced) and 160 mussels of *M. chilensis* (native, from Chile) were analysed. Regarding COI gene variation in *M. galloprovincialis*, 35 nucleotide sites were polymorphic, and 23 were parsimony-informative, and a total of 38 haplotypes were identified. For *M. chilensis*, 69 haplotypes and 76 polymorphic sites were identified, 25 of which were parsimony-informative. For 16S variation in *M. galloprovincialis* from Spain, South Africa and Chile, 20 polymorphic sites, 12 parsimony-informative sites, and 20 haplotypes were identified. For *M. chilensis,* 70 polymorphic sites, 16 parsimony-informative sites, and 49 haplotypes were identified.

The haplotypes of both species were deposited in GenBank under the accession numbers MZ357027-40; MZ313467; MZ313219-21 (*M. galloprovincialis*); MZ356925-7015; and MZ313212-18 (*M. chilensis*).

### 3.2. Genetic Structure and Gene Lineages

For the COI gene data, Geneland, which takes into account spatial information, indicated *K* = 2 as the most likely population structure, with the detected genetic groups (*M. chilensis* versus *M. galloprovincialis*) being geographically clustered (Figure 1). The assignment probabilities of individuals to their respective clusters were ≥0.90 (Figure 1).

**Figure 2:**
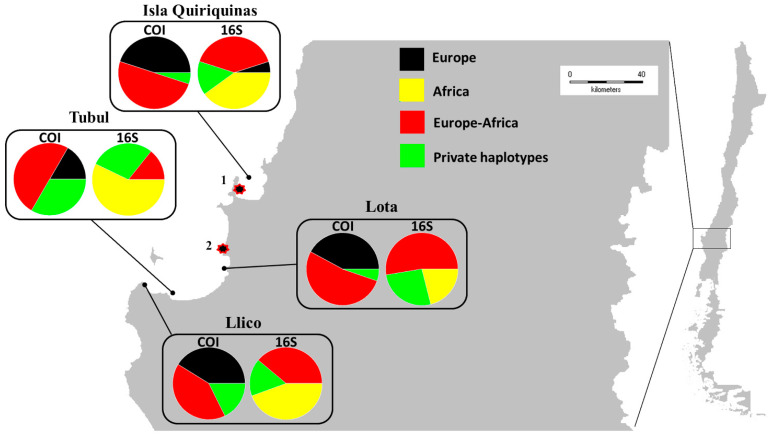
Distribution of invasive populations of *Mytilus galloprovincialis* in Chile (i.e., the northern group from the Geneland analysis, Figure 1). 1 = Puerto Talcahuano; 2 = Puerto Coronel (see details of the sites in Table 1). Code in colour/regional analysis: black/Europe—haplotypes found in Chilean mussels that were previously reported only in European mussels; red/Europe–Africa—haplotypes found in Chilean mussels that were previously reported only in European and/or African mussels; yellow/Africa—haplotypes found in Chilean mussels that were previously reported only in African mussels; green/private haplotypes—haplotypes found only in mussels from Chile.

Between 20% (16S) and 15% (COI) of the haplotypes found in *M. galloprovincialis* from Chile were private, and the rest of them were shared with mussels from Europe and/or South Africa (Figure 2). There was no evidence of a genealogical pattern amongst the localities analysed; only a network of haplotypes without structure was apparent (Figure S2). On the other hand, *M. galloprovincialis* from Chile showed a genetic distance of 1.6% in relation to mussels from South Africa and of 1.1% in relation to mussels from Europe.

For the COI gene in *M. chilensis*, one haplotype (H4) was found in every population and occurred at high frequency (19.3%). Excluding H4, 10 other haplotypes were shared by at least three localities. These shared haplotypes represented 60% of the total number of individuals. Finally, 59 haplotypes were private (unique to a single population), most of them being singleton haplotypes (Figure S2). Few dominant haplotypes were observed for the COI gene, which indicated a model of recent population expansion, since haplotypes shared amongst localities were commonly observed, representing a recent genetic connection event. For the 16S sequence data, the same trend was observed, although with more numerically dominant haplotypes (Figure S2).

### 3.3. Genetic Diversity

For *M. galloprovincialis* from Chile, haplotypic diversity varied between 0.832 and 0.906 (COI) and between 0.754 and 0.905 (16S). However, the highest haplotypic diversity was recorded in the invasive population of South Africa [COI = 1 (SD: 0.016); 16S = 0.742 (SD: 0.071)]. For *M. chilensis*, haplotypic diversity varied between 0.914 and 0.971 (COI) and between 0.678 and 0.879 (16S) (see Table 2). Based on multispecies comparisons [99], the nucleotide diversity values (π) were high for both species. However, the values of π and Π were higher for *Mytilus galloprovincialis* (up to 3.9 times for π and 3.7 times for Π) than for *M. chilensis*. Nevertheless, the highest values of nucleotide diversity and average number of nucleotide differences were recorded for non-native *M. galloprovincialis* from South Africa (Table 2).

### 3.4. Demographic Analyses

The global Tajima’s *D* and Fu’s *Fs* neutrality tests were both negative and statistically significant for the entire COI and 16S datasets, except for the samples from Spain and South Africa (Table 2).

**Figure 3:**
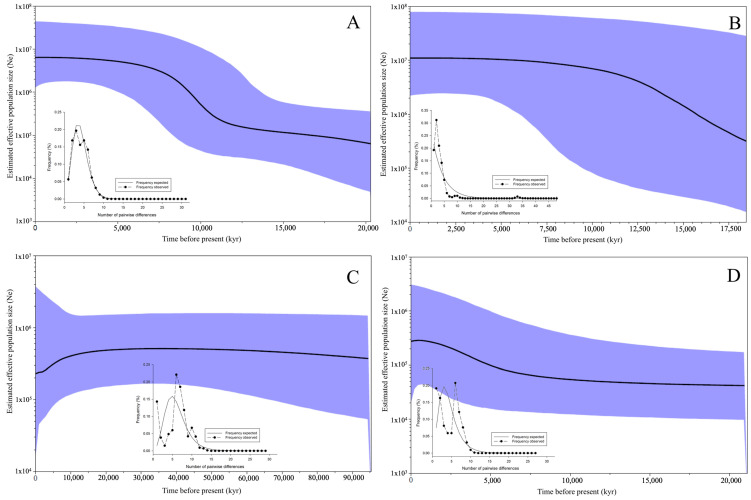
Bayesian skyline plots (BSPs) and mismatch analysis (MA). BSPs showing the demographic history of *Mytilus chilensis* ((**A**) = COI; (**B**) = 16S) and *M. galloprovincialis* ((**C**) = COI; (**D**) = 16S) along the Chilean coast. The dark lines represent median values for the population size (Ne); the blue area marks the 95% highest probability density intervals in all panels. The MA provided the frequency distribution of pairwise differences among the haplotypes.

As expected for the star-like network (Figure S2) of *M. chilensis*, the mismatch distribution for COI was L-shaped (Figure 3A). The Bayesian skyline plot analysis showed an increase in the effective population size 19,000 years ago, with a tendency to balance (reach equilibrium) ~3750 years ago. The same trend was observed for the 16S locus, which showed a pulse of population expansion 18,000 years ago (Figure 3B).

For COI variation in *M. galloprovincialis* in Chile, a constant effective population size was observed ~90,000 years ago, followed by a slight decrease in the present (Figure 3C). On the other hand, for 16S, the effective population size was constant for ~15,000 years and then slightly increased (Figure 3D). The mismatch distribution analysis indicated an adjustment to a population stasis model, due to the fact that the histogram showed an observed multimodal curve, both for COI and for 16S (Figure 3C,D).

### 3.5. Phylogenetic Relationships

**Figure 4:**
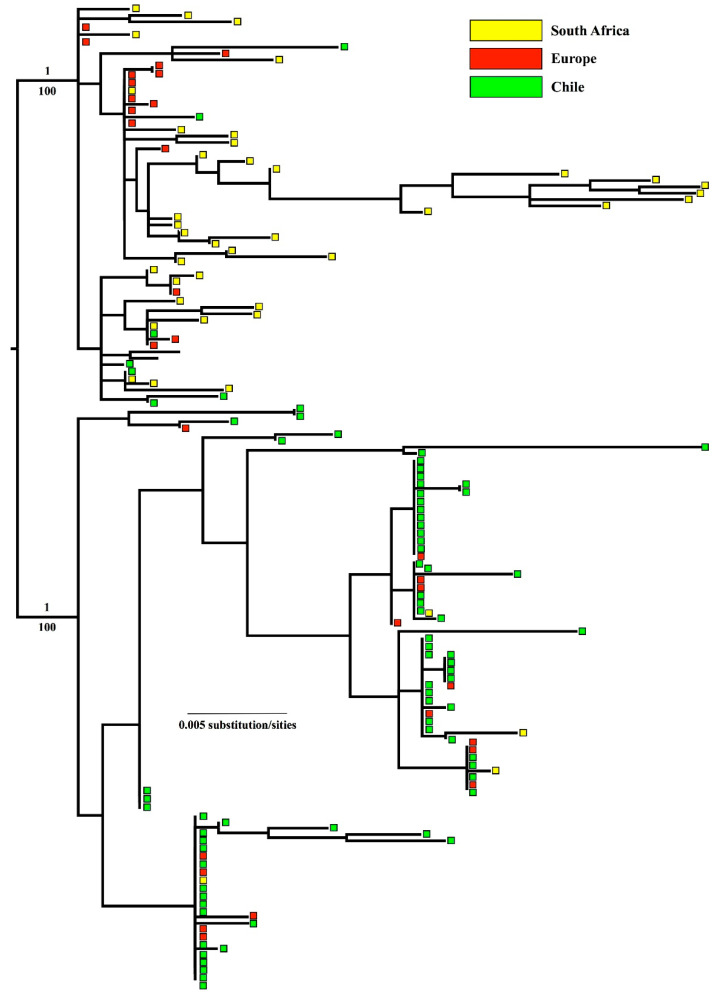
Maximum likelihood tree of the concatenated dataset (COI+16S) of *M. galloprovincialis* from Chile, Spain and South Africa (Table 1) and sequences from GenBank (Table S2). Bayesian analysis returned the same topology. Numbers above the branches are Bayesian posterior probabilities/maximum likelihood bootstrapping values.

Testing for the best fit substitution model resulted in the selection of the models TrN+I+G (1st, best fit), TrN+G (2nd) and TVNef+I (3rd) for COI and TrN+I for 16S. The BI and ML analyses revealed almost identical topologies, with two major clades being strongly supported. Clade 1 (BPP = 1/BS = 100) mainly included animals from Europe and South Africa, and clade 2 (BPP = 1/BS = 100) included mussels from Chile and Europe (Figure 4).

A discrete phylogeographic analysis revealed that the majority of Chilean mussels belonged to the Mediterranean lineage (see Figure S3). In addition, a divergence and an almost reciprocal monophyly were revealed between the Mediterranean lineage and the Atlantic lineage, represented by the mussels from Spain and South Africa. The divergence between the lineages occurred ~235,000 years ago (Figure S3).

## 4. Discussion

### 4.1. Populations of Mytilus spp. in Chile

An extensive survey was carried out to establish the distribution of blue mussels along the Chilean coast, from 30° S (Tongoy) in the north to 53° S (Caleta Pescadores) in the south. Mussels were only found at sites south of 36° S, but not with a continuous distribution. Two genetically distinct groupings of *Mytilus* were detected, although this genetic differentiation was not correlated with the biogeographical break described for the native biota of the Chilean coast between 41° and 48° S [100,101]. Specifically, between 38° and 42° S (without *Mytilus* presence), the coast has exposed sandy beaches of different morphodynamic types that alternate with intertidal sandbanks at the mouths of rivers [102]. This results in a natural, large spatial-scale mosaic of different habitat types, many of which are not suitable environments for blue mussels [103], which may act as a natural break (i.e., extensive habitat discontinuity) in the range of an otherwise continuously distributed species.

Regarding the distribution of the invasive *M. galloprovincialis* in Chile, we observed a limited spread: it is only located within ~198 km of coastline between Isla Quiriquinas (36°37′ S) and Llico (37°10′ S) (see Figure 1 and Figure 2). However, the distribution of this species within this region is not continuous (e.g., it is missing from Chivilingo and Laraquete): it is normally associated with rocky environments, where it interacts with other endemic intertidal mussels (i.e., *Choromytilus chorus*, *Semimytilus patagonicus* (Hanley, 1843) and *Perumytilus purpuratus* (Lamarck, 1819); Oyarzún PA, personal observation). Recently, Díaz-Puente et al. (2020) [68] sampled blue mussels along the Chilean coastline from Pelluco (41°30′ S; 72°53′ W) in the Los Lagos region (total of four sites) to Puerto Williams (54°56′ S; 67°36′ W) in the Magallanes region (a total of six sites). Using a range of RFLP and microsatellite markers, they too failed to detect *M. galloprovincialis* in their sampling range. Thus, there is presently no evidence that *M. galloprovincialis* in Chile has spread very far from its likely point of introduction (see below).

On the other hand, the native Chilean mussel (*M. chilensis*) has a broad distribution, from 38°47′ S to 54°57′ S, covering approximately 2100 km of the Chilean coast [9,31,35,68]. It is an ecologically important species, as well as the basis for a large and economically important aquaculture industry [104,105]. However, this native species exhibited very low genetic differentiation amongst the natural beds sampled along its whole distribution range. In other studies, a low genetic differentiation between natural beds of Chilean mussels was also reported (e.g., [106,107,108,109]). This may be due to the aquaculture of this mussel that involves the transfer of juveniles (seeds) from collection areas to the growth centres located in bays and fjords with high primary production [110]. This suggestion is confirmed by the recent findings of Díaz-Puente et al. (2020) [68] based on their microsatellite analysis of four mussel populations from the Los Lagos region and six populations from the Magallanes region. They reported a clear separation of the four northerly (Los Lagos) from the six southerly (Magallanes) populations of *M. chilensis* and attributed this to 50 years of aquaculture enhancement in Los Lagos (a region that they termed a ‘genetic hodgepodge’), in contrast to the ‘pristine’ situation in the Magellanic region, where there is little or no aquaculture.

### 4.2. Invasion of Chile by Mytilus Galloprovincialis

*M. galloprovincialis* is thought to have only recently invaded the Pacific coast of South America [47]. In the 1990s, artisanal fishermen called these mussels “choro araucano”, the name being derived from the geographical area where they live (choro araucano = mussels of the region of Araucania). Our demographic results (Bayesian skyline plots) revealed that the population size of *M. galloprovincialis* in Chile has decreased, contrary to what was seen for *Mytilus chilensis*, which supports the suggestion of a recent appearance of the Mediterranean mussel on the Chilean coast.

In Chile, the Mediterranean lineage of *M. galloprovincialis* is the dominant form (85%), and the Atlantic lineage is less common (15%). These results, therefore, suggest multiple introductions of *Mytilus galloprovincialis* to Chile. Earlier, Larraín et al. (2018) [33] used SNP markers to highlight the Mediterranean origin of *M. galloprovincialis* at Cocholgue (35°7′38.62″ S; 73°11′25.56″ W) and the absence of North Atlantic *M. galloprovincialis* in the same general region as that of our samples. In the present study, the region where the invasive mussels were found is the location of two of the main ports in Chile (Talcahuano and Coronel), both of which serve as centres for ship traffic with Europe [111]. Our collection sites of Tubul and Lota are located near the presumptive introduction epicentre (i.e., near the two ports), given that the mussels from these reef sites showed the greatest number of private haplotypes (over 30% of the haplotypes). Tubul and Lota are located ~10 km south of Coronel port within the Gulf of Arauco, and these sites have rocky substrates that are favourable environments for the settlement and establishment of blue mussels. Further sampling within the port environments and also at other sites near to the ports (e.g., [55])may help elucidate if one port or the other was the primary point of entry for one or both lineages of *M. galloprovincialis* and may improve our understanding of the progression of their spread from the port to natural sites.

High genetic diversity was observed for *Mytilus* spp. in Chile, above the average values reported for marine invertebrates [99]. Values are considered to be high when haplotypic diversity is >0.70130 and nucleotide diversity is >0.00356 [99]. However, and somewhat surprisingly, the greatest genetic diversity was found in South African mussels (mostly *M. galloprovincialis* of the North Atlantic lineage; Zardi et al., 2018 [61]), coincidentally the country where they have had the greatest invasion success in the intertidal ecosystem [57,58,112]. We need to study more closely the relationship between biological invasion success and genetic diversity to determine just how critical genetic diversity is for invasive species. Regardless, it is increasingly clear that some aquatic/marine invasions do not exhibit the reduced levels of genetic diversity that might be expected in small founder populations but instead exhibit surprisingly high levels of genetic diversity (the “paradox” of Roman and Darling 2007 [23]). Given the commercial nature of the ports of Talcahuano and Coronel, it seems more than likely that this region of Chile has experienced multiple separate invasion events, via hull fouling and/or ballast water discharge (different vessels from different ports of origin at different times), which will themselves tend to promote haplotypic diversity in *M. galloprovincialis* within Chile. The ML tree revealed two separate lineages of *M. galloprovincialis*, with the North Atlantic lineage dominating in the South African mussels, and the Mediterranean lineage dominating in the Chilean mussels. Thus, whilst the invasive *M. galloprovincialis* populations in South Africa and Chile are, apparently, derived from different regions of Europe, there is evidence of both lineages (but at very different proportions) in South Africa and Chile. These general findings are in line with reports of invasive *M. galloprovincialis* in other Southern Hemisphere countries, with both lineages being present in Australia and New Zealand, where the Mediterranean lineage dominates (e.g., [7,36,38,41]), but not in South Africa, where the North Atlantic lineage dominates [61].

### 4.3. Spread of M. galloprovincialis in Chile

There is general agreement that once established at a few or multiple sites, there is no real prospect of successfully eradicating invasive *M. galloprovincialis* (e.g., [7,8,9,56]). Once an invasive species has arrived and established (i.e., pre-border management options have not been successful in preventing its entry into a country), then the best course of action is to use post-border options such as internal borders to slow down or, at best, prevent the further spread of the invader (e.g., [113]). Internal borders may take different forms and can include, for example, long stretches of coastline of unsuitable habitat that form a natural distributional break and/or a barrier to the species spread, including larval dispersal. In Chile, as noted above, to the north of the epicentre of *M. galloprovincialis* introduction, the habitat in the region between 42° and 38° S is largely unsuitable for *Mytilus* spp. [103] and may therefore act as a natural internal border that slows or possibly prevents the northern spread of *M. galloprovincialis*. Internal borders may also include natural biogeographic breaks or transition zones (e.g., [59,113,114]). At the moment, *M. galloprovincialis* in Chile is restricted in its distribution to the Araucanian region (region 178 in Spalding et al., 2007 [115]). Even if the region of unsuitable habitat between 38° and 42° S were breached, then further northward *M. galloprovincialis* spread might be limited by the transition from the Araucanian to the Central Chile region (region 177 in Spalding et al., 2007 [115]). To the south, the spread of *M. galloprovincialis* may also be slowed or halted at the biogeographic boundary between the Araucanian region and the Chiloense region (region 188 in Spalding et al., 2007 [115]). Recently, Ma et al. (2021) [59] showed how such natural biogeographic boundaries may act as weak or strong barriers to the spread of invasive *M. galloprovincialis* in South Africa. However, they noted that whilst such natural borders may act as barriers to this species spread, at least temporally, on occasions, when the boundaries are breached, a subsequent spread could be rapid [59]. It is still not clear when or why a biogeographic boundary may act as a barrier to this species further spread, but it seems likely that a change in environmental conditions (e.g., strong and localised upwelling, regional sea surface temperature and/or salinity differences) and/or differences in substratum type and habitat availability may be important.

## 5. Conclusions

The Chilean biodiversity and protected areas agency categorised *M. galloprovincialis* as a species of “major concern” [116]. For this reason, the Undersecretary of Fisheries and Aquaculture (SUBPESCA) commissioned an evaluation and prospecting study of this mussel in the Chilean territory. Toro et al. (2017) [67] suggested permanent monitoring that includes the use of molecular tools for diagnosing its presence/absence and not allowing its cultivation because it could endanger the native marine biodiversity. In Chile, the best management options to control the spread of *M. galloprovincialis* now appear to be (1) enhanced ship-based monitoring and surveillance (i.e., pre-border or border inspections) to prevent further introductions, given that we know that many invasions are not based on a single event but come from several or multiple different sites and/or regions across multiple years, (2) the development of a regional monitoring programme to quantify *M. galloprovincialis* spread within the Araucanian region, and (3) monitoring of locations, in particular ports and marinas, identified as being at the highest risk of introductions, to look for post-border within-country transfers (e.g., [54]). If found at such sites, a successful eradication programme could reasonably be mounted (e.g., [117,118]). However, for this strategy to be successful, there have to be ongoing monitoring (to detect incursions as soon as possible) and the political will and in-field ability to respond urgently.

## Figures and Tables

**Table 1:** Site survey information, number of specimens collected (*n*), and geographical coordinates of the sites.

Species and Country/Site	Map Code	Coordinates	*n*
*M. galloprovincialis*			
CHILE			
Isla Quiriquinas	IQ	36°37′49.9″ S; 73°03′09.2″ W	20
Lota	LT	37°04′27.1″ S; 73°09′53.4″ W	20
Tubul	TU	37°13′37.3″ S; 73°26′02.2″ W	20
Llico	LL	37°10′06.9″ S; 73°33′41.5″ W	20
SPAIN			
Moaña-Pontevedra	MP	42°16′28.5″ N; 8°43′48.3″ W	20
SOUTH AFRICA			
Port Elizabeth	PE	33°58′52.0″ S; 25°39′36.0″ E	20
*M. chilensis*			
CHILE			
Puerto Saavedra	PS	38°46′44.7″ S; 73°24′32.3″ W	20
Chaihuín	CH	39°56′40.4″ S; 73°34′40.4″ W	20
Maullín	MA	41°37′25.3″ S; 73°35′36.9″ W	20
Quillaipe	QL	41°32′59.5″ S; 72°45′14.0″ W	20
Huildad	HU	43°03′02.8″ S; 73°34′21.1″ W	20
Puerto Cisnes	PC	44°44′11.4″ S; 72°41′07.9″ W	20
Estero Fanny	BF	53°05′04.6″ S; 72°18′39.6″ W	20
Caleta Pescadores	CP	53°21′06.2″ S; 70°57′27.8″ W	20
Total number of mussels			280

**Table 2:** Diversity indices and tests of neutrality for *M. galloprovincialis* and *Mytilus chilensis* based on data for COI (676 bp) and 16S (470 bp) sequence variation. K = number of haplotypes; H = haplotypic diversity; S = number of polymorphic sites; Π = mean number of pairwise differences; π = nucleotide diversity; Tajima’s *D* = Tajima’s *D* test; Fu’s *F*_S_ = Fu’s *F*_S_ test; ns = not significant.

Species/Locality	K		H (SD)		S		Π		π		Tajima’s *D*		Fu’s *F*_S_	
	COI	16S	COI	16S	COI	16S	COI	16S	COI	16S	COI	16S	COI	16S
*M. galloprovincialis*														
CHILE (introduced)														
Isla Quiriquina	7	8	0.832 (0.049)	0.826 (0.061)	31	13	10.66	3.821	0.01669	0.00832	0.58	0.16	5.14	−0.10
Lota	7	8	0.865 (0.042)	0.754 (0.090)	28	11	8.392	3.094	0.01349	0.00674	0.05	−0.36	3.57	−0.89
Tubul	9	5	0.889 (0.049)	0.905 (0.103)	21	9	6.157	4.190	0.00980	0.00913	−0.15	0.74	0.24	−0.05
Llico	12	6	0.906 (0.050)	0.810 (0.057)	34	10	10.60	3.007	0.01754	0.00655	−0.20	0.12	−0.22	0.68
All locations	14	16	0.857 (0.020)	0.809 (0.035)	28	18	5.065	3.384	0.00863	0.00737	−0.45	−0.48	0.50	−3.28
SPAIN (native)														
Moaña-Pontevedra	6	7	0.757 (0.091)	0.872 (0.067)	25	10	7.191	3.538	0.01124	0.00751	ns	ns	ns	ns
SOUTH AFRICA (introduced)														
Port Elizabeth	20	7	1.000 (0.016)	0.742 (0.071)	50	10	11.84	2.116	0.01830	0.00449	ns	ns	ns	ns
*M. chilensis*														
CHILE (native)														
Puerto Saavedra	14	9	0.947 (0.038)	0.678 (0.122)	20	16	3.257	2.292	0.00484	0.00488	−1.87	−2.11	−8.43	−2.93
Chaihuín	13	9	0.942 (0.037)	0.678 (0.122)	15	14	2.532	1.936	0.00375	0.00412	−1.68	−2.05	−8.37	−3.64
Maullín	14	8	0.971 (0.032)	0.860 (0.055)	19	8	3.632	1.721	0.00539	0.00366	−1.39	−0.97	−9.04	−3.23
Quillaipe	12	7	0.932 (0.035)	0.824 (0.064)	20	8	3.789	1.662	0.00561	0.00354	−1.25	−1.05	−3.96	−2.15
Huildad	10	8	0.914 (0.056)	0.867 (0.067)	14	38	2.895	5.590	0.00428	0.01189	−1.30	−2.28	−4.31	−0.14
Puerto Cisnes	15	9	0.942 (0.043)	0.879 (0.043)	21	9	3.647	1.932	0.00540	0.00411	−1.46	−1.10	−8.89	−3.46
Bahía Fanny	14	10	0.947 (0.034)	0.711 (0.113)	21	14	3.089	1.816	0.00457	0.00386	−1.82	−1.98	−8.28	−5.01
Caleta Pescadores	15	11	0.921 (0.055)	0.763 (0.103)	28	10	3.326	1.442	0.00492	0.00307	−2.26	−1.90	−9.63	−8.16
All locations	69	49	0.943 (0.011)	0.808 (0.030)	76	70	3.161	2.289	0.00470	0.00488	−2.48	−2.64	−94.83	−59.09

## Data Availability

All data generated or analysed during this study are included in this published article (and its Supplementary Materials files). Furthermore, the DNA sequences are stored in Genbank.

## Supplementary Materials

The following supporting information can be downloaded at: https://www.mdpi.com/article/10.3390/ani14060823/s1, Table S1. Sites where *Mytilus* spp. are absent on the Chilean coast; Table S2. GenBank accession numbers and sample site information for sequence data used for this study for cytochrome *c* oxidase subunit I (COI); Figure S1. Plot of the number of clusters (*K*) simulated from the posterior distribution obtained with Geneland for the analysis of Chilean mussel genetic diversity; Figure S2. mtDNA (COI and 16S) maximum parsimony network analyses including sequences of *Mytilus galloprovincialis* and *M. chilensis* from different localities in Chile. Each haplotype is represented by a coloured circle indicating the main location where it was collected. Circle sizes are proportional to haplotype frequencies. The numbers represent the minimum number of mutational steps between haplotypes; Figure S3. Maximum clade credibility tree from the Bayesian analyses based on cytochrome oxidase subunit I (COI) sequences for *Mytilus galloprovincialis* lineages. The colour of the branches corresponds to the most probable location of their descendant nodes. The main lineages were Mediterranean (red) and Atlantic (fuchsia). The same colour coding is presented in the pie charts at key nodes. The sequences used are presented in Table 1 and Table S2. References [119,120] are cited in the Supplementary Materials.
