# Supplementary material for: The Mediterranean Mussel Mytilus galloprovincialis (Mollusca: Bivalvia) in Chile: Distribution and Genetic Structure of a Recently Introduced Invasive Marine Species
Source: Animals (Basel). 2024 Mar 7;14(6):823. doi: 10.3390/ani14060823 (PMC10967297; doi:10.3390/ani14060823)
Supplement: Supplementary file 1 [file animals-14-00823-s001.zip › animals-2821084-supplementary.pdf]

Supporting information

Table S1. Sites where *Mytilus* spp. are absent on the Chilean coast.

Site	Inspection date	Coordinates
Tongoy	07.02.2016	30°15’22.33’’S;71°30’10.85’’W
Pelluhue	26.01.2016	35°48’48.36’’S;72°34’40.82’’W
Curanipe	26.01.2016	35°50’32.52’’S;72°38’14.54’’W
Pullay	26.01.2016	36°01’06.93’’S;72°46’44.65’’W
Buchupureo	26.01.2016	36°04’38.23’’S;72°47’23.63’’W
Cobquecura	26.01.2016	36°08’05.73’’S;72°47’40.15’’W
Caleta Taucu	26.01.2016	36°10’53.75’’S;72°48’57.79’’W
Colmuyao	25.01.2016	36°11’44.18’’S;72°49’24.31’’W
Purema	25.01.2016	36°26’42.28’’S;72°52’55.67’’W
Merquiche	25.01.2016	36°29’07.94’’S;72°54’23.66’’W
Puda	25.01.2016	36°30’00.06’’S;72°54’29.81’’W
Isla Mocha	14.03.2016	38°23’32.32’’S;73°54’34.21’’W
Chivilingo	17.04.2016	37°08’37.66’’S;73°01’17.60’’W
Laraquete	17.04.2016	37°10’27.43’’S;73°11’57.84’’W
Caleta Ránquil	05.12.2015	37°31’00.00’’S;73°37’00.00’’W
Tirúa	05.12.2015	38°20’28.68’’S;73°30’07.17’’W

Table S2. GenBank accession numbers and sample site information of sequence data used for this study for cytochrome *c* oxidase subunit I (COI).

**Sample location**	**GenBank Accession**	***N***	**Reference**
Samos, Greece	AY130054 to AY130060	7	Riginos et al. (2004) [119]
Chioggia, Italy	AM905220 to AM905225	6	[39]
Paternoster Bay, South Africa	AM905216 to AM905219	4	[39]
South Africa	DQ351477 to DQ351497	20	[120]

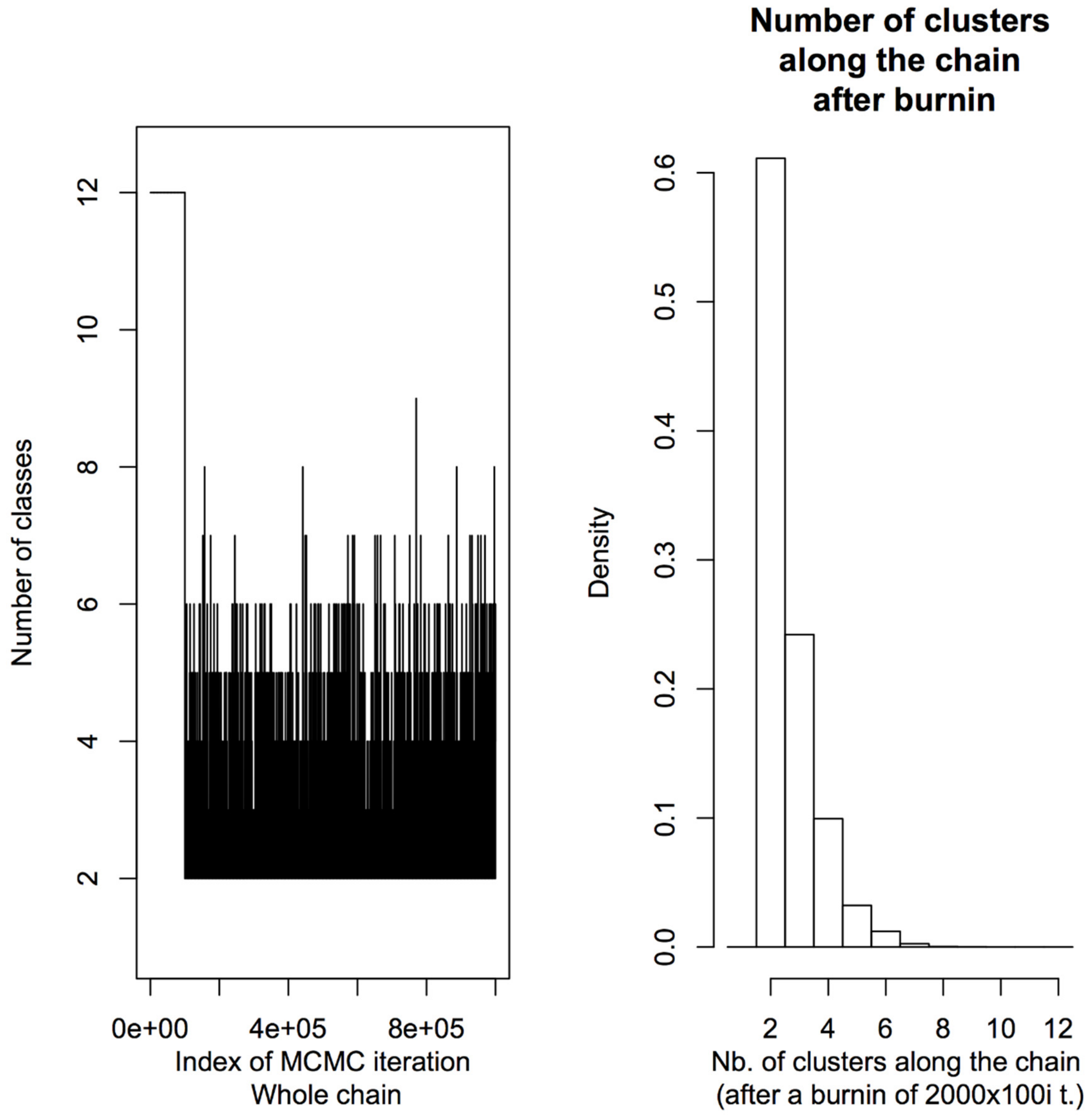

Figure S1. Plot of the number of clusters (*K*) simulated from the posterior distribution obtained with Geneland for the analysis of Chilean mussel genetic diversity.

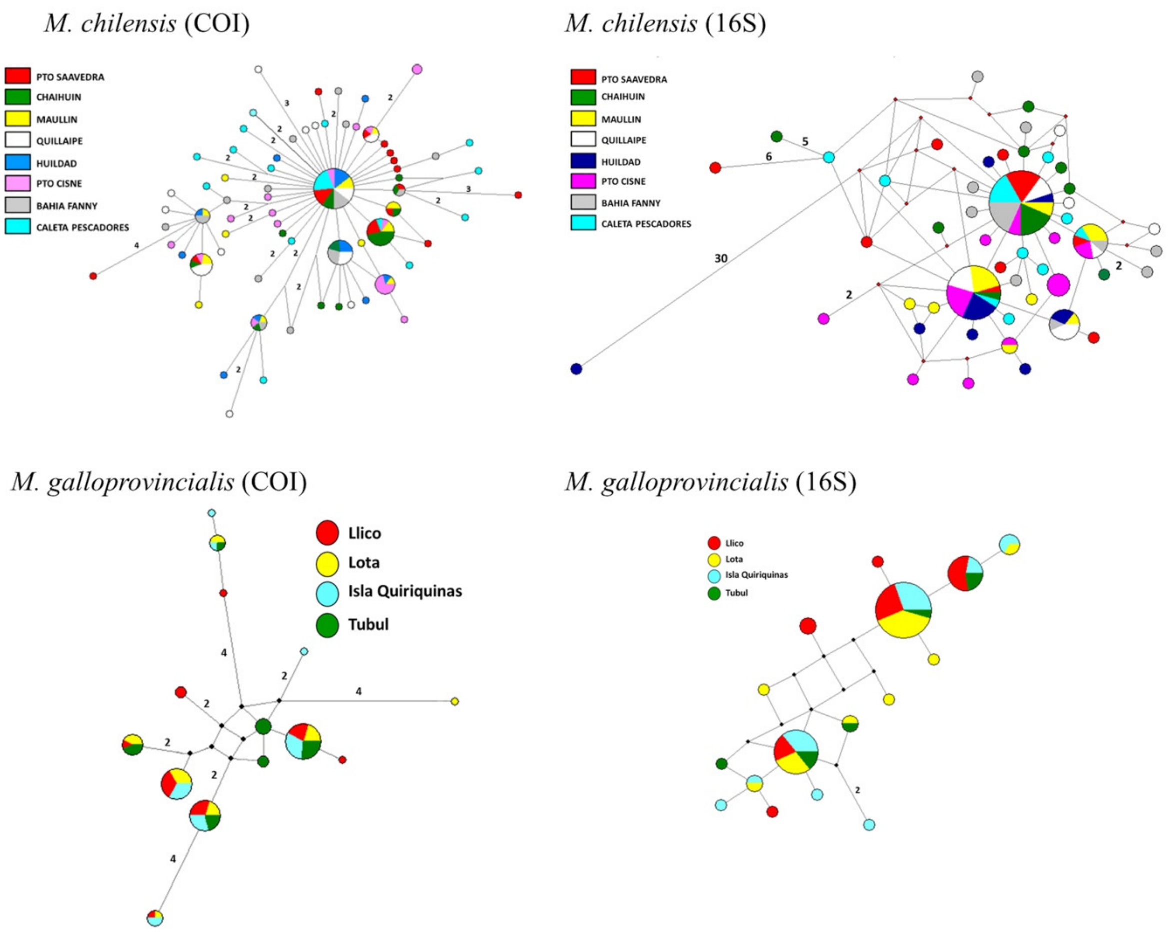

Figure S2. mtDNA (COI and 16S) Maximum Parsimony network analyses including sequences of *Mytilus galloprovincialis and M. chilensis* from different localities in Chile. Each haplotype is represented by a coloured circle indicating the main location where it was collected. Circle sizes are proportional to haplotype frequencies. The numbers represent the minimum number of mutational steps between haplotypes.

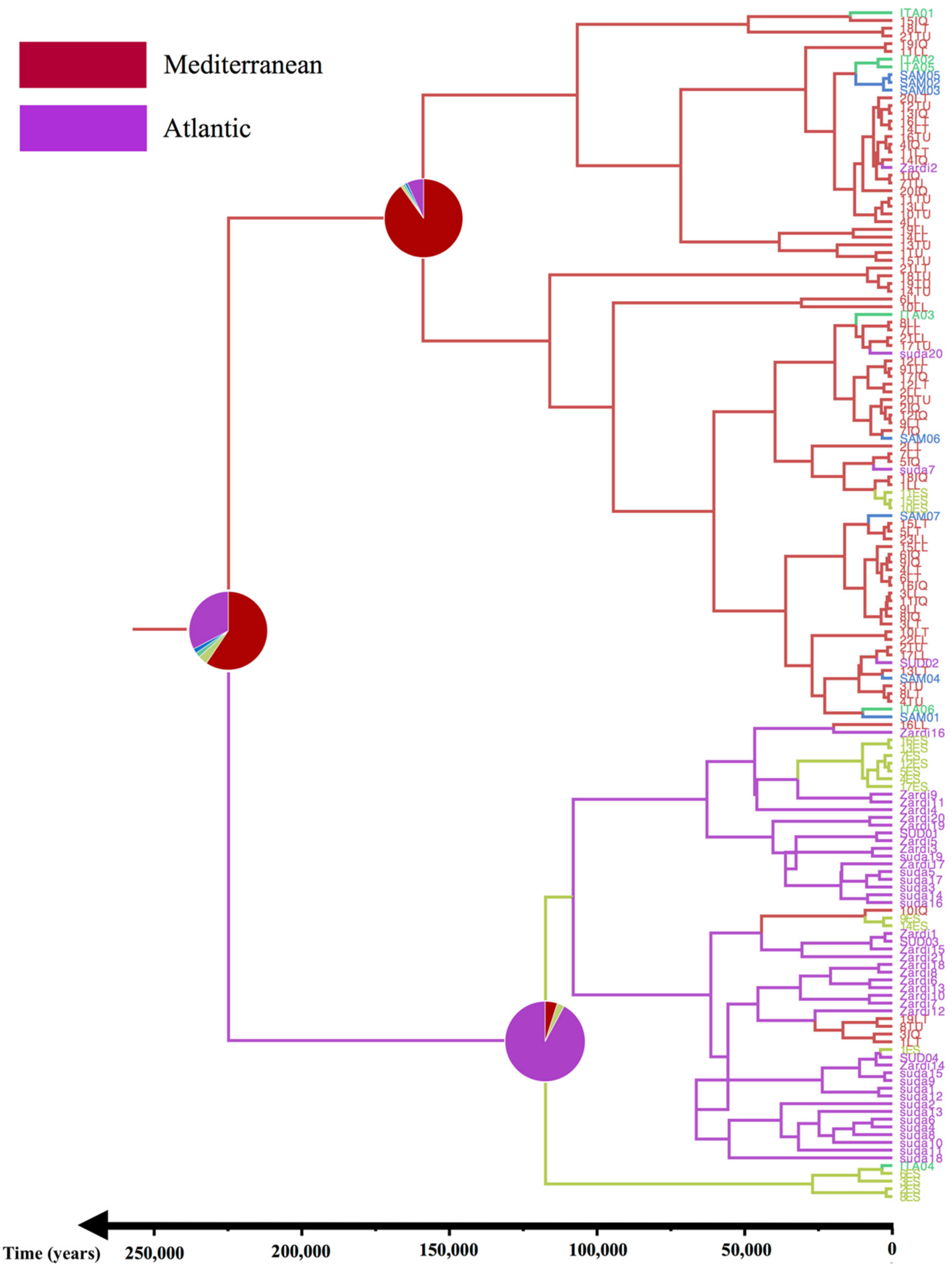

Figure S3. Maximum clade credibility tree from the Bayesian analyses based on Cytochrome Oxidase subunit I (COI) sequences for *Mytilus galloprovincialis* lineages. The colour of the branches corresponds to the most probable location of their descendant nodes. The main lineages were Mediterranean (red) and Atlantic (fuchsia). The same colour coding is presented in the pie charts at key nodes. The sequences used are presented in Tables 1 and S2.
